# Molecular and neuronal plasticity mechanisms in the amygdala-prefrontal cortical circuit: implications for opiate addiction memory formation

**DOI:** 10.3389/fnins.2015.00399

**Published:** 2015-11-05

**Authors:** Laura G. Rosen, Ninglei Sun, Walter Rushlow, Steven R. Laviolette

**Affiliations:** ^1^Addiction Research Group, Schulich School of Medicine and Dentistry, University of Western OntarioLondon, ON, Canada; ^2^Department of Anatomy and Cell Biology, Schulich School of Medicine and Dentistry, University of Western OntarioLondon, ON, Canada; ^3^Department of Psychiatry, Schulich School of Medicine and Dentistry, University of Western OntarioLondon, ON, Canada

**Keywords:** opiates, addiction, memory, dopamine, amygdala, prefrontal, molecular

## Abstract

The persistence of associative memories linked to the rewarding properties of drugs of abuse is a core underlying feature of the addiction process. Opiate class drugs in particular, possess potent euphorigenic effects which, when linked to environmental cues, can produce drug-related “trigger” memories that may persist for lengthy periods of time, even during abstinence, in both humans, and other animals. Furthermore, the transitional switch from the drug-naïve, non-dependent state to states of dependence and withdrawal, represents a critical boundary between distinct neuronal and molecular substrates associated with opiate-reward memory formation. Identifying the functional molecular and neuronal mechanisms related to the acquisition, consolidation, recall, and extinction phases of opiate-related reward memories is critical for understanding, and potentially reversing, addiction-related memory plasticity characteristic of compulsive drug-seeking behaviors. The mammalian prefrontal cortex (PFC) and basolateral nucleus of the amygdala (BLA) share important functional and anatomical connections that are involved importantly in the processing of associative memories linked to drug reward. In addition, both regions share interconnections with the mesolimbic pathway's ventral tegmental area (VTA) and nucleus accumbens (NAc) and can modulate dopamine (DA) transmission and neuronal activity associated with drug-related DAergic signaling dynamics. In this review, we will summarize research from both human and animal modeling studies highlighting the importance of neuronal and molecular plasticity mechanisms within this circuitry during critical phases of opiate addiction-related learning and memory processing. Specifically, we will focus on two molecular signaling pathways known to be involved in both drug-related neuroadaptations and in memory-related plasticity mechanisms; the extracellular-signal-regulated kinase system (ERK) and the Ca^2+^/calmodulin-dependent protein kinases (CaMK). Evidence will be reviewed that points to the importance of critical molecular memory switches within the mammalian brain that might mediate the neuropathological adaptations resulting from chronic opiate exposure, dependence, and withdrawal.

## Introduction

The persistent and compulsive behavioral patterns associated with opiate addiction depend largely on the ability of opiate-class drugs to produce powerful associative memories linked to their intense, euphorigenic properties. Indeed, the ability of environmental cues to trigger opiate-related associative memories is arguably the primary driver of the addiction cycle, characterized by repeated cycles of chronic use, withdrawal and ultimately, relapse. While many classes of abused drugs share the ability to elicit rewarding effects and dependence, opiate-class drugs, whether illicit or prescribed, stand alone in their ability to not only evoke intensely subjective experiences of pleasure, but also in terms of their capacity to produce both severe physiological and psychological withdrawal effects. The severity of these opiate-related effects is illustrated not only by the exponentially increasing and global spread of opiate addiction, with recent epidemiological reports suggesting approximately 15.5 million people worldwide (Degenhardt et al., 2014), but also by the well-established and documented clinical intractability of opiate dependence.

Decades of both clinical and basic neuroscience research have revealed many underlying neurobiological mechanisms responsible for the motivational properties of opiates. However, the neural processes responsible for the encoding, recall, and extinction of opiate-related drug reward memories have only recently begun to be understood. For example, how do early, acute drug-experiences become encoded in the brains emotional memory circuits? Once chronic exposure and dependence have developed, do separate neuronal or molecular memory substrates come online during the processing of addiction-related memory? Do these neural memory adaptations lead to increased resistance and/or increased motivational salience linked to drug-associated cues? Adding to the complexity of addiction-related memory processing is the recognition that distinct phases of learning and memory involve different molecular and neuroanatomical substrates. Drug-related associative memories must first be encoded and consolidated via associative neuronal and molecular mechanisms. These drug-related memories must then be stored in terms of their temporal and contextual information. Finally, recall of these associative memories can serve as triggers, responsible for compulsive drug seeking and relapse, even many years after successful abstinence has been established.

Elucidating the neural mechanisms responsible for the formation and persistence of opiate-related associative memories has the potential for opening up new therapeutic vistas for both the prevention and/or reversal of the profound effects on neuronal learning and memory plasticity underlying the development of opiate dependence. In particular, understanding the underlying molecular pharmacological substrates linked to these phenomena may reveal potential new pharmacotherapeutic targets for the treatment of protracted opiate dependence and/or relapse. In this review, we will examine recent evidence implicating the mammalian prefrontal cortex (PFC) and basolateral amygdala (BLA) as critical neural regions involved in the control of opiate-related addiction memory phenomena. Specifically, we will examine how functional interactions not only within the BLA-PFC circuit, but also with associated mesocorticolimbic dopamine (DA) signaling, may control the processing of opiate-addiction related associative memories. As will be described, emerging evidence is suggesting that opiate exposure state represents a functional boundary between distinct molecular and neuronal mechanisms within this circuitry, mediating how the brain acquires, recalls, and ultimately may extinguish, addiction-related memories.

## Neural mechanisms underlying the encoding of associative opiate reward memories

Opiate class drugs, either illicit or prescribed, are capable of producing potent rewarding effects which quickly lead to states of physiological (somatic) and/or psychological dependence. Given the well-documented euphorigenic properties of opiates, decades of research have explored the primary neural regions and pathways responsible for these acute reinforcing effects and how the mammalian brain encodes the environmental and/or internal cues associated with these phenomena. Importantly, considerable evidence has demonstrated that the neural mechanisms responsible for these acute, rewarding properties can be dissociated from those mediating the secondary effects of opiates, such as the formation of drug-related associative memories and/or withdrawal-related motivational and somatic phenomena. In this context, the mesolimbic DA pathway comprising the ventral tegmental area (VTA) and its associated DAergic (A10) neuronal projections to the nucleus accumbens (NAc) has consistently been identified as the most critical neural region underlying the acute rewarding stimulus properties of opiates, in both humans and other mammals (Wise, 1989; Wise and Rompre, 1989; Nader and van der Kooy, 1997; Koob and Volkow, 2010). For example, rats will directly self-administer opiates or related derivatives into the VTA (David and Cazala, 1994; Devine and Wise, 1994; Steidl et al., 2015) and microinfusions of morphine into the VTA produce powerful associative reward memories measured in place conditioning procedures (CPP; Nader and van der Kooy, 1997; Olmstead and Franklin, 1997; Bishop et al., 2011; Lintas et al., 2011). Interestingly, infusions of opiates into regions extrinsic to the VTA but which share direct or indirect functional connections, such as the basolateral amygdala (BLA), caudate putamen, medial prefrontal cortex (mPFC), hippocampus, lateral hypothalamus, pedunculopontine tegmental nucleus (PPT), ventral pallidum or nucleus accumbens (NAc), generally fail to produce primary reinforcing behavioral effects (Olmstead and Franklin, 1997).

Nevertheless, while the acute rewarding properties of opiates may depend primarily upon the VTA, several of the neural regions noted above are capable of modulating the reinforcing effects of opiates. For example, excitotoxic lesions of the brainstem PPT are capable of abolishing both operant opiate self-administration (Olmstead and Franklin, 1994; Olmstead et al., 1998) and opiate reward measured in Pavlovian associative conditioning procedures such as the conditioned place preference paradigm (CPP), which involves conditioning an experimental animal in either a drug-paired or vehicle-paired environment and then testing whether the animal develops a conditioned preference for the environment previously paired with the effects of the drug (Bechara and van der Kooy, 1989; Nader and van der Kooy, 1997; Bechara et al., 1998). Lesions of the rat PFC, specifically within the infralimbic (IFL) sub-region, have been reported to block morphine CPP reward (Tzschentke and Schmidt, 1999). Furthermore, opiate receptor transmission in the VTA has been shown to modulate electrical brain self-stimulation reward thresholds measured in the ventral pallidum (Panagis et al., 1998).

Importantly, a substantial body of literature demonstrated that blockade of the mesolimbic DA system, either via neurochemical lesioning with 6-hydroxydopamine (6-OHDA) or via administration of DA receptor antagonists, were able to strongly attenuate the primary reinforcing properties of opiates, measured in a variety of behavioral paradigms, including intravenous self-administration (IVSA) and CPP (Wise, 1989; Wise and Rompre, 1989). However, subsequent reports demonstrated that the functional role of DA transmission in the context of opiate-reward processing, depended crucially upon the relative opiate exposure state of the animal. Thus, a significant number of reports demonstrated that while the acute rewarding effects of opiates could be mediated through non-DAergic neural mechanisms, once animals became chronically exposed to opiates such as morphine or heroin, the reinforcing effects of opiates became dependent upon DA transmission (Bechara and van der Kooy, 1989; Nader and van der Kooy, 1997; Bechara et al., 1998). As will be discussed below, these studies laid the foundation for characterizing both DA-dependent and independent neural motivational pathways within the mesolimbic system, and how opiate exposure states may control the processing of opiate reward behaviors through distinct neural circuits.

## Exposure state determines which neural pathways control the primary rewarding effects of opiates: evidence for an opiate addiction “switch?”

A critical consideration when investigating or theorizing about the neural mechanisms controlling the motivational effects of opiates, is the organism's prior history of drug exposure. For example, theoretical addiction frameworks focusing exclusively on drug-related withdrawal phenomena, cannot account for the acute effects of drug motivation nor the mechanisms responsible for the initiation of drug use. Similarly, theoretical frameworks focusing on drug-related sensitization or similar plasticity mechanisms within neural reward pathways such as the mesolimbic system, do not account for specific mechanisms controlling the switch from the non-addicted to addicted neural states or potential mechanisms underlying the reversibility of addiction-related plasticity. The importance of identifying discrete neural switching mechanisms controlling the transition from non-dependent to drug-dependent motivational states is particularly important in the context of opiate addiction. Indeed, even a single exposure to high concentrations of opiates such as morphine are capable of inducing acute states of dependence and/or withdrawal (Larcher et al., 1998; Kawasaki et al., 2011; Rothwell et al., 2012), highlighting the potentially rapid and fluid nature of the opiate addiction process.

A considerable body of basic neuroscience research has indicated that the state of opiate exposure (e.g., previously naïve, dependent, or dependent and in a state of withdrawal) can regulate dissociable neural motivational circuits and mechanisms controlling opiate reward processing. For example, Bechara et al. (for review see Bechara et al., 1998) reported a functional dissociation between two separate and dissociable neural motivational pathways sub-serving the rewarding properties of morphine, as a function of exposure and withdrawal state. Thus, while excitotoxic lesions of the brainstem PPT only blocked morphine reward CPP in previously opiate-naïve rats, these same lesions were ineffective once rats were made opiate-dependent and were conditioned in a state of drug withdrawal. In contrast, systemic blockade of DA receptor transmission with a broad-spectrum DA receptor antagonist failed to block opiate reward effects in opiate-naïve rats, but completely blocked morphine CPP when rats were conditioned while in a state of opiate-dependence and withdrawal. This functional dissociation was later localized directly to the VTA; thus, intra-VTA morphine reward was shown to depend on the DA-independent, PPT neural motivational system in the opiate naïve state, but switched to a DA-dependent substrate once rats were conditioned in the dependent and withdrawn states (Nader and van der Kooy, 1997). This apparent switch in brain reward signaling from the naïve to dependent/withdrawn states was later replicated in rats performing heroin IVSA (Olmstead et al., 1998), demonstrating that regardless of Pavlovian or Operant conditioning paradigms, the primary rewarding effects of opiates could be dissociated between a drug-naïve, DA-independent system, and a DA-dependent system once chronic exposure and withdrawal was present (Nader and van der Kooy, 1997; Bechara et al., 1998; Laviolette et al., 2002).

The molecular mechanisms underlying this switch were later characterized as involving an alteration in the excitability properties of VTA GABA_A_ receptors, associated with non-DAergic, presumptive GABAergic neurons (Laviolette and van der Kooy, 2001; Laviolette et al., 2004). Thus, in opiate-naïve rats, it was found that pharmacologically blocking inhibitory VTA GABA_A_ receptors activated a DA-independent behavioral reward signaling pathway (mediated through the brainstem PPT; Laviolette and van der Kooy, 2004). However, once rats were made opiate-dependent and were in a state of withdrawal, VTA GABA_A_ receptors switched to an excitatory signaling state, and now activated a DA-dependent reward signaling pathway (mediated through the mesolimbic DA system). However, while these intra-VTA opiate exposure plasticity mechanisms may account for the primary rewarding effects of opiates, critical questions remained. For example, how might opiate exposure state control neural memory substrates controlling the associative memory effects of opiates? Do the neural substrates processing these powerful, opiate-related associative memories similarly depend upon a plasticity gradient, controlled by the brain's state of opiate dependence or withdrawal? Two neural regions sharing substantial functional connectivity with the VTA and associated DAergic transmission, are the BLA and PFC. Indeed, a large body of evidence from both human and animal-based opiate addiction models has pointed to a role for these neural regions as important candidates mediating the processing of associative memories linked to opiate reward and withdrawal behaviors.

## Encoding of opiate reward memory in the amygdala-prefrontal cortical pathway

The mammalian VTA and mesolimbic pathway share many important functional connections with both cortical and sub-cortical brain regions involved in emotional processing and associative memory formation. Two regions in particular that have been implicated in opiate-related learning and memory are the PFC and BLA; both of which share functional connections with each other, and receive important DAergic inputs from the VTA. Evidence for the role of the PFC in both human and animal-based models of opiate addiction come from a variety of imaging, behavioral, and molecular studies. For example, functional imaging studies have demonstrated that the activation of PFC regions and associated cortical circuitry is correlated with the expression and/or cue-induced recall of associative memories linked to opiate-taking experiences in human opiate-dependent subjects (Daglish et al., 2001; Luo et al., 2004; Volkow et al., 2004; Langleben et al., 2008; Yang et al., 2009). While human imaging studies are necessarily limited in terms of anatomical and circuit-level resolution, evidence from animal models of opiate addiction have revealed important roles for neuronal networks within the PFC during the encoding, recall, and extinction of opiate-related associative memories.

**Figure 1:**
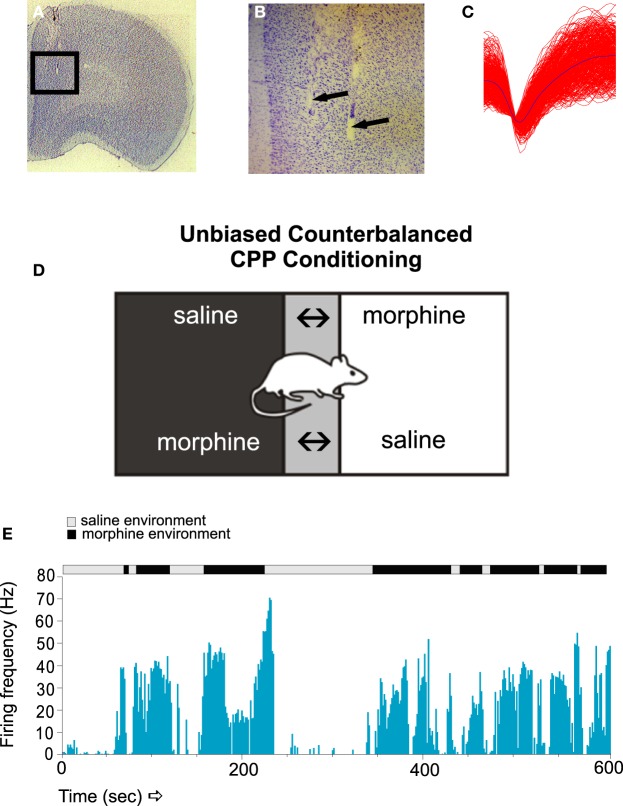
**The encoding of associative opiate reward memories in PFC neuronal populations**. **(A,B)** Microphotographs showing representative microwire recording electrodes in the rat PFC. **(C)** A sample *in vivo* recording waveform showing a typical PFC neuronal trace. **(D)** Unbiased place conditioning (CPP) involves randomly pairing the subject with a vehicle saline injection in one distinct environment, vs. a drug-injection (e.g., morphine) in an alternate conditioning environment. Following multiple associative training sessions, animals are then asked to choose which environment to spend time in during the recall test, as a behavioral measure of associative memory learning. If the animal recalls the rewarding effects of morphine, it will display a preference for the environment previously paired with the rewarding properties of morphine. **(E)** A sample raster recording of a PFC neuron during a morphine CPP recall test. PFC neurons display robust associative increases in firing, specifically in response to exposure to the previously experienced morphine-paired environments. Figure Adapted from Sun et al. (2011).

For example, using chronically implanted micro-wire recording arrays in the prelimbic cortex (PLC) of the rat, Sun et al. (Figures 1A–C; Sun et al., 2011), reported that sub-populations of PLC neurons were capable of encoding opiate-related reward memories measured in a Pavlovian conditioned place preference procedure (Figure 1D). Thus, specific PLC neuronal subpopulations showed strong associative neuronal activity, both in terms of firing frequency and bursting levels, specifically during exposure to conditioned environmental cues linked to the rewarding effects of morphine (Figure 1E). Interestingly, PLC neuronal sub-populations demonstrated specific patterns of firing activity depending upon the phase of opiate reward memory processing (e.g., acquisition, recall or extinction of the memories). Furthermore, detailed analysis of these associative PLC neuronal activity patterns revealed several differences between the firing modes of these cortical neurons; for example, whether neurons were firing in regular, “tonic” patterns vs. bursting in irregular spike trains. PLC neurons were found to display divergent activity patterns of bursting activity during the initial acquisition (e.g., memory encoding) vs. the extinction (i.e., forgetting) of associative opiate reward memories, with cortical neuron bursting activity selectively increasing during the extinction phase of opiate-related memory processing. Such findings were consistent with previous evidence showing that neuronal populations in the rat PFC were capable of showing differential response patterns during different phases of opiate-related drug seeking behaviors (Chang et al., 1997). Furthermore, evidence for a role of PFC neuronal ensemble activation during reinstatement of extinguished heroin seeking was demonstrated by Shalev et al. (2003), who reported that reinstatement of heroin self-administration induced by acute food deprivation could selectively induce c-fos activation in PLC neuronal populations. In addition, Schmidt et al. (2005) demonstrated that even after 3 weeks of heroin extinction training and abstinence, cue-induced heroin self-administration (but not reinstatement of sucrose seeking) in rats was correlated with selective increases in zif268 (a molecular marker of early gene activation) immunoreactivity in selective PLC neuronal subpopulations. Together, such evidence suggests that, similar to human imaging studies, prefrontal cortical regions are critical for the storage and cue-related recall of associative opiate-reward memories. However, considerable evidence has pointed to the involvement of sub-cortical associative memory regions such as the BLA as being important players in the processing of opiate-related memories, particularly in terms of shared functional connectivity with cortical regions.

Similar to effects described in the PFC, the BLA has been reported to play a critical role in the processing of various forms of opiate-related associative memories. Interestingly, despite receiving substantial VTA DAergic inputs (Ford et al., 2006), the BLA itself appears to play no role in the mediation of opiate-related primary reinforcement effects (Olmstead and Franklin, 1997). Rather, the functional role of the BLA as an interface between VTA DAergic inputs and outputs to other cortical and limbic regions (such as the PFC and NAc), position this heterogeneous structure to sub-serve associative memory functions. Indeed, single neurons within the BLA can encode emotionally salient associative memories via the convergence of DAergic inputs with sensory-associative information (Grace and Rosenkranz, 2002; Rosenkranz and Grace, 2002). Not surprisingly, the BLA has been implicated in the associative properties of opiate-related learning and memory. For example, pharmacological inactivation of the BLA has been shown to abolish cue or heroin-induced reinstatement of extinguished heroin-seeking behaviors in rats (Fuchs and See, 2002). Beyond a role in processing reward-related properties of opiates, the expression of opiate-withdrawal related aversion memories has been linked to specific VTA-BLA neuronal activation patterns. Using c-fos as a molecular marker of neuronal activation patterns within the VTA>BLA network, Frenois et al. (2005) reported that while BLA neuronal activation was involved in the associative memory processing of opiate withdrawal, neurons within the adjacent central nucleus (CeA) were preferentially activated during acute withdrawal, independently of conditioning, suggesting a more selective role for the BLA in associative learning and memory components of opiate-related conditioned behaviors. Converging evidence from clinical studies has further implicated the amygdala as an important component of opiate-related cue processing. For example, Xie et al. (2011) reported that impulsivity measures in heroin-abstinent subjects were correlated with hyperactivity in an extended amygdala-frontal cortical network. Thus, convergent evidence from both animal and clinical based studies point to a significant role for the BLA in both the storage and expression of associative memories linked to both the acute and long-term effects of opiate exposure. Furthermore, considerable evidence points to important functional connectivity between the BLA and PFC during the processing of opiate-related associative information.

## Evidence for functional BLA-PFC interactions in associative opiate-related addiction memories

The BLA and PFC share critical functional and anatomical relationships and through bi-directional efferent and afferent connections. In terms of the functional role of the BLA-PFC pathway in human heroin addiction, little is known in terms of molecular or neuronal mechanisms. However, in addition to fMRI studies discussed previously, heroin dependent individuals have been reported to display disturbances in both resting state connectivity and functional activity relationships in the BLA-PFC network (Zhang et al., 2011, 2015). Nevertheless, considerable evidence from animal models of opiate reward processing has demonstrated important functional interconnections between the BLA and PFC during both the acute acquisition and the consolidation of associative opiate reward memories. For example, Bishop et al. (2011) demonstrated that pharmacologically inducing a state of NMDA receptor hypofunction in the rat PFC was able to strongly potentiate the reward salience of normally sub-reward threshold conditioning doses of morphine, measured in a CPP procedure. Interestingly, this effect was dependent upon active BLA inputs to the PFC, since pharmacologically inactivating the BLA prior to PFC modulation of NMDA transmission was sufficient to block PFC-mediated modulation of opiate reward memory potentiation.

**Figure 2:**
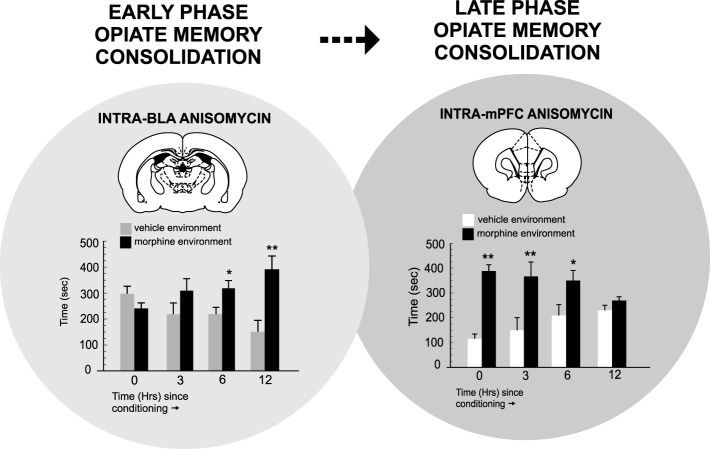
**The temporal dynamics of opiate reward memory consolidation within the BLA → PFC pathway**. Targeted protein synthesis inhibition with anisomycin within the BLA administered immediately after associative morphine CPP training (consolidation of memory phase) blocks associative opiate reward memory consolidation only during the early phase of consolidation (0–6 h post-training). In contrast, targeting the PFC revealed that PFC-dependent opiate reward memory consolidation occurred only during later-phase consolidation (6–12 h post-training). ^*^p < 0.05; ^**^p < 0.01. Figure Adapted from Gholizadeh et al. (2013).

**Figure 3:**
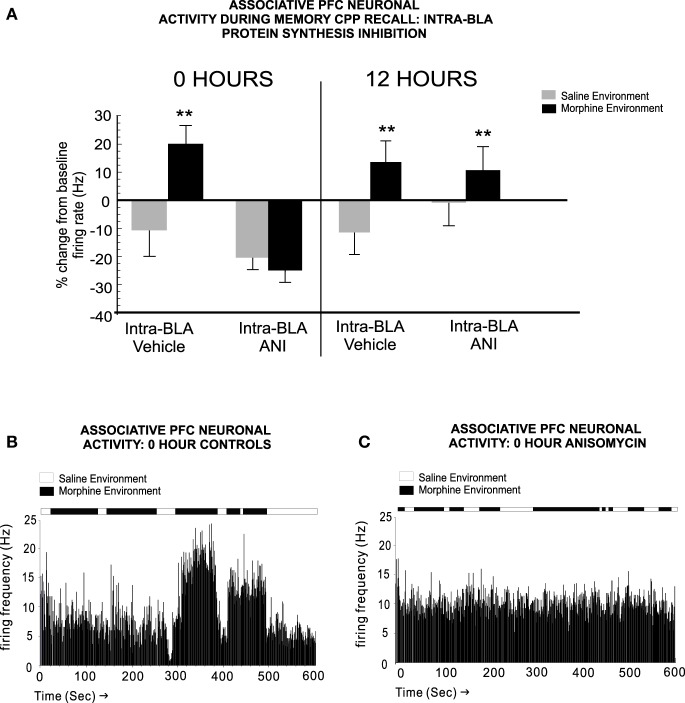
**Blockade of intra-BLA associative opiate reward memory consolidation prevents the transfer of long-term opiate reward memories to the PFC**. **(A)** Blocking protein synthesis in the BLA at 0 h post-CPP training, prevents associative increases in PFC neuronal firing rates when tested 24 h later. At 12 h, associative memory is already transferred to PFC neuronal populations and associative neuronal responses are present during the CPP recall test phase. **(B)** A sample rastergram showing a typical PFC neurons associative response to morphine-environment exposure during the recall test, from a rat receiving intra-BLA vehicle control infusions at 0 h post-training. **(C)** In contrast, blocking BLA protein synthesis at 0 h post-training with anisomycin, completely blocks associative neuronal responding during the recall test phase, in this representative neuronal rastergram. ^**^p < 0.01. Figure Adapted from Gholizadeh et al. (2013).

In terms of PFC neuronal involvement in the temporal consolidation of opiate-related associative reward memories, BLA inputs are critical. For example, Gholizadeh et al. (2013) used targeted protein synthesis inhibition in the BLA → PFC pathway to demonstrate that the acute, early consolidation phase of an associative morphine-reward memory was dependent upon the BLA. However, during later phase consolidation, PFC-dependent memory consolidation substrates were brought online. Interestingly, this anatomically dissociable transfer of opiate reward consolidation substrates along the BLA-PFC pathway was dependent upon post-learning temporal dynamics. Thus, BLA-dependent memory consolidation was only required during the first 0–6 h post-conditioning and was mediated through an extracellular-signal-regulated-kinase (ERK) molecular substrate. In contrast, during later phases of memory consolidation (6–12 h post-training), a PFC-dependent, calcium-calmodulin-kinase-α, ERK-independent memory substrate was activated. These findings demonstrated that during later phases of addiction-related memory formation, PFC memory substrates are specifically brought online, following a period of first-order memory consolidation in sub-cortical limbic regions, such as the BLA (Figure 2). Next, using microwire recordings of PFC neurons combined with intra-BLA protein synthesis inhibition at early vs. late phase consolidation of acute opiate associative reward memory, Gholizadeh et al. (2013) demonstrated that intra-BLA inhibition of associative opiate reward memory consolidation, blocked associative PFC neuronal responses during later CPP recall testing (Figure 3), further demonstrating the ability of intra-BLA associative opiate reward memory consolidation mechanisms to control remote consolidation in PFC neuronal sub-populations.

Further demonstrating the importance of BLA inputs to the PFC during associative opiate memory formation, reversible inactivation of the BLA during opiate-related reward learning was shown to increase the spontaneous firing and bursting activity of rat PFC neurons and alter the longer-term memory processing associated with opiate-related conditioning effects. For example, Sun and Laviolette (2012) demonstrated that pharmacologically inactivating the rat BLA during the acquisition phase of opiate reward CPP conditioning causes a later acceleration in the extinction of previously learned opiate reward memories reflected in associative PFC neuronal activity changes during conditioning. Thus, removing BLA input to the PFC during associative opiate reward memory formation, led to a less stable encoding of the memory which was more vulnerable to extinction. Together, these findings demonstrate that specific sub-populations of neurons in the mammalian PFC are capable of differentially encoding specific phases of addiction-related memories and are governed by relatively precise temporal phases of drug-reward exposure and active inputs from consolidation mechanisms in the BLA. Furthermore, such evidence implicates an important functional role for PFC neurons not only in the initial acquisition of addiction-related associative memories, but also in the later consolidation and recall of these memories.

## Opiate exposure state regulates dopamine receptor function in the basolateral amygdala: implications for associative opiate memory formation

In human heroin users, functional dysregulation between amygdala and cortical cue reactivity networks has been correlated with heightened sensitivity to drug-related cues (Liu et al., 2009a). In terms of BLA involvement in the processing of reward-related associative memory, DAergic inputs from the VTA to the BLA have been demonstrated to modulate both opiate-related motivational signaling and BLA neuronal activity dynamics (Kröner et al., 2005; Ford et al., 2006). Nevertheless, until recently, the underlying pharmacological and molecular substrates underlying the functional role of the BLA in opiate-related associative memory formation were not well understood.

The anatomical and functional position of the BLA as a recipient of DAergic VTA inputs and location of functional outputs regulating both NAc and PFC neuronal populations (Floresco et al., 2001; Ford et al., 2006; Tan et al., 2011; Lintas et al., 2012) suggests that neurons in the BLA may function as an associative memory interface regulating opiate memory formation. However, the precise roles of specific DA receptor subtype transmission in the control of opiate memory formation have only recently been elucidated. Using targeted microinfusions of selective DA D1 vs. D2 receptor antagonists in rats, Lintas et al. (2011) demonstrated that transmission through DA receptors directly in the BLA was able to potently control the formation of associative morphine reward memories as a function of opiate exposure state. Using an unbiased place conditioning procedure to measure the rewarding properties of morphine, the authors reported that while intra-BLA blockade of the DA D1 receptor (D1R) with a selective D1R antagonist, prevented the formation of opiate reward memories only in rats that were conditioned in a previously opiate-naïve state, once rats were chronically exposed to opiates and conditioned in a state of withdrawal, the intra-BLA DA receptor substrate responsible for the processing of associative morphine reward memories switched to a DA D2 receptor (D2R)-dependent substrate. This DA D1R/D2R receptor-dependent switch was dependent upon downstream activation or inhibition of the cyclic AMP (cAMP) signaling pathways as simultaneous intra-BLA co-administration of a cAMP inhibitor reversed the behavioral effects of D1 receptor blockade on morphine CPP in the opiate-naïve state. In contrast, in rats trained in a state of opiate-dependence and in withdrawal, intra-BLA co-administration of a cAMP activator, was shown to reverse the ability of intra-BLA D2 receptor blockade to prevent opiate reward memory formation. In addition, the ability of BLA D1R/D2R receptor transmission to control opiate reward memory formation was demonstrated both for the processing of systemically applied morphine, and for processing the direct, rewarding effects of intra-VTA morphine infusions, demonstrating a functional link between VTA → BLA DAergic projections in the mediation of opiate related associative memory acquisition (Lintas et al., 2011).

**Figure 4:**
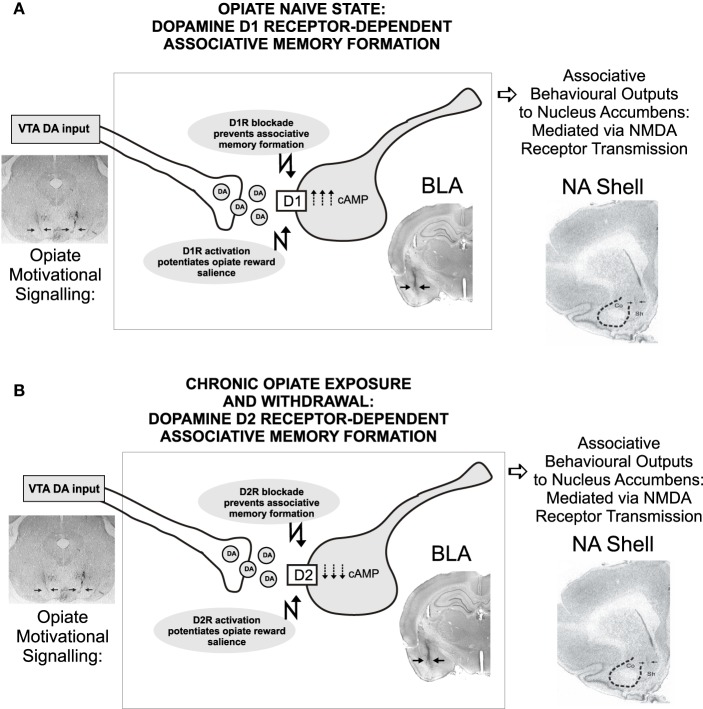
**Transmission through dopamine D1 vs. D2 receptor substrates in the rat BLA control the formation of associative opiate reward memories as a function of opiate exposure state**. Thus, in rats conditioned in a previously opiate-naïve state **(A)**, blockade of intra-BLA D1 receptors dose-dependently blocked the formation of associative opiate reward memories measured in a CPP procedure, through a cAMP-dependent molecular mechanism. In contrast, once chronic opiate exposure and withdrawal has occurred **(B)**, DA-dependent BLA opiate reward memory switches to a D2 receptor-dependent, cAMP mechanism, independently of D1 transmission, as blockade of intra-BLA D2 transmission dose-dependently blocks opiate reward memory formation selectively in rats chronically exposed to opiates and conditioned in a state of withdrawal. Figure Adapted from Lintas et al. (2011).

In follow-up studies, it was reported that intra-BLA transmission through either DA D1 or D2 receptor substrates could control not only the acquisition of associative opiate reward memories, but also regulate the motivational salience of opiate-related reward memories via functional interactions with the NAc. Thus, infusions of either selective D1R or D2R agonists directly into the BLA was shown to strongly increase the reward salience of normally sub-reward threshold conditioning doses of morphine, again, measured in a place conditioning procedure (Lintas et al., 2012). Similar to the effects of intra-BLA D1R/D2R blockade on opiate reward memory formation, the effects of *activating* intra-BLA D1R/D2R substrates on morphine-reward salience potentiation were dependent upon the opiate exposure state of the animal; D1R activation only was effective in previously opiate-naïve subjects, while D2R activation was only effective when conditioning took place following chronic opiate exposure and in the presence of withdrawal, again demonstrating a functional transition between the D1R vs. D2R BLA receptor systems as a function of opiate exposure state (Figure 4).

Given the well-established importance of the NAc in processing of opiate-related reward memories and evidence that the BLA can strongly regulate neuronal activity in the NAc via excitatory BLA → NAc inputs (Wise, 1989; Wise and Rompre, 1989; Floresco et al., 2001). Lintas et al. (2012) next examined the potential role of BLA → NAc projections in mediating the effects of intra-BLA D1R/D2R receptor transmission on opiate reward salience. They reported that blocking NMDA receptor transmission selectively in the shell region of the NAc (NASh) was sufficient to block the opiate reward potentiating effects of intra-BLA DA receptor activation. Interestingly, these behavioral effects were correlated with the observation that intra-BLA D1R or D2R activation could strongly potentiate NASh neuronal responses to morphine administration, however, in line with the previously observed behavioral dissociation, BLA DA receptor activation was demonstrated to follow the same functional boundary between the opiate-naïve vs. chronic exposure and withdrawal states. Thus, similar to the effects of D1R/D2R blockade, the BLA has been demonstrated to control the motivational salience of opiate reward memory formation via direct interactions with NASh neuronal populations (Lintas et al., 2012).

The demonstration of a localized, intra-BLA D1R-D2R memory switching mechanism as a function of opiate exposure is consistent with previous reports demonstrating that chronic opiate exposure and withdrawal may selectively induce sensitization of D2R-dependent motivational pathways. An early *in vitro* study using the isolated guinea pig ileum as a model for opiate withdrawal, demonstrated that D2R agonists and antagonists selectively controlled naloxone-induced tissue contracture effects, while D1R agents showed little efficacy (Capasso and Sorrentino, 1997). From a behavioral perspective, Druhan et al. (2000), in a series of systemic behavioral pharmacology experiments, demonstrated that rats displayed heightened sensitivity to the locomotor activating effects of D2R agonists specifically during spontaneous opiate withdrawal. In contrast, D1R agonists had no effects. Similarly, Harris and Aston-Jones (1994) demonstrated that direct activation of D2R within the NAc, was capable of attenuating somatic and the aversive behavioral effects of opiate withdrawal. Thus, while these studies did not directly examined associative opiate reward memory effects, they are nevertheless suggestive of a general chronic opiate-induced mechanism, leading to a switch between D1R to D2R-dependent physiological and behavioral substrates controlling the transition from the naïve, to the dependent and withdrawn opiate exposure states.

Nevertheless, important questions remain. For example, what are the underlying molecular substrates linked to changes in the functional properties of BLA D1/D2 receptors in the modulation of opiate-related addiction memories? How might chronic exposure to opiates induce this functional memory switch within the VTA-BLA-NAc-PFC circuitry? As will be discussed presently, considerable evidence now suggests that chronic opiate exposure may control molecular memory adaptations within the BLA-PFC circuitry via alterations in the phosphorylation states of extracellular-signal-regulated kinases (ERK) and calcium-calmodulin-kinase (CaMK) signaling families. Furthermore, these adaptations may be functionally linked to intra-BLA D1R/D2R regulation of opiate reward memory processing.

## Molecular memory substrates controlling opiate memory formation: role of ERK 1–2 and CaMKIIα-dependent memory plasticity

The molecular underpinnings of associative, drug-related memory formation, particularly at the neuronal and synaptic levels, are gradually beginning to be elucidated. Two neuronal signaling pathways linked to DA receptor transmission and which have been associated with drug-related reward processing and memory formation are the extracellular-signal-regulated kinases 1 and 2 (ERK1/2) and calcium/calmodulin-dependent protein kinase II-α (CaMKIIα) systems. Evidence for the roles of these pathways in drug-related learning and memory come from various lines of evidence. For example, exposure to drug-related conditioning cues using rodent models of drug self-administration or associative conditioning has been repeatedly shown to modulate ERK expression levels in a variety of neural regions linked to reward-related processing, including the amgydala, PFC and NAc (Lu et al., 2000, 2005; Mizoguchi et al., 2004; Miller and Marshall, 2005; Valjent et al., 2006; Girault et al., 2007; Lin et al., 2010; Li et al., 2011).

In the amygdala, dynamic ERK signaling events are associated with various stages of drug-related memory processing. For example, Wells et al. (2012) demonstrated that intra-BLA ERK activation in the BLA was essential for processing of cocaine-related associative memory reconsolidation. Li et al. (2008) reported that ERK phosphorylation in the CeA was critical for the incubation of opiate-related drug craving behaviors, wherein the intensity of opiate-related associative drug-seeking memory and behavior was demonstrated to increase throughout an extended withdrawal period. Furthermore, the recall of associative opiate-related reward memories has been linked to ERK phosphorylation events in the rat BLA (Li et al., 2011).

The CaMKIIα signaling pathway has similarly been associated with various forms of drug-related memory processing. For example, reinstatement of extinguished opiate-seeking behaviors occurs in association with increased phosphorylation of CaMKIIα at threonine 286 in the NAc shell but not core sub-regions (Liu et al., 2012a). Furthermore, pharmacological blockade of CaMKII signaling in the NAc shell blocks reinstatement of morphine self-administration behaviors (Liu et al., 2012b). Chen et al. (2008) reported that long-term, but not acute exposure to morphine led to upregulation in CaMKII mRNA levels selectively in the hippocampus and PFC. In contrast, long-term exposure to the synthetic heroin substitute, methadone, was reported to downregulate phosphorylated levels of CaMKIIα in the rat hippocampus, further suggesting that chronic opiate exposure is capable of functionally modifying levels of CaMKII (Andersen et al., 2012).

Importantly, in terms of DAergic transmission, signaling through both D1R and D2R has been linked to downstream modulation of ERK1/2 and CaMKIIα pathways. However, considerable evidence suggests dissociable roles for D1R vs. D2R subtypes their respective modulatory effects. Thus, activation of D1R substrates within the mesocorticolimbic pathway has been associated with phosphorylation of the ERK 1/2 signaling cascade. For example, administration of D1R agonists has been reported to increase phosphorylation levels of ERK selectively in the NAc and PFC, while D2 agonists had no effect (Xue et al., 2015). In terms of reward-related behavioral processing, Kirschmann et al. (2014) reported that the ability of reward-related conditioned stimuli to activate ERK signaling in the NAc, was mediated through a D1R-NMDA dependent mechanism. Bertran-Gonzalez et al. (2008) reported that psychostimulant-induced activation of ERK phosphorylation in the NAc was confined exclusively to D1R-containing striatal neurons, providing yet further evidence for functional D1R-mediated selectivity over downstream modulation of ERK phosphorylation events, at least in the context of drug-related effects in the mesolimbic system.

In contrast, regulation of D2R transmission has been linked to downstream modulation of the CaMKII-signaling cascade in various behavioral and molecular assays. First, at the cellular level, D2R function is functionally linked to calcium signaling. For example, the neuronal calcium sensor-1 (NCS-1) has been shown to control desensitization of the D2R (Kabbani et al., 2002; Woll et al., 2011), suggesting that ambient calcium levels may importantly regulate D2R function. In terms of drug-related modulation of D2R-CaMKII signaling, Liu et al. (2009b) demonstrated that in NAc neurons, CaMKIIα is recruited to the D3 receptor subtype (a member of the D2R family which shows high expression levels in mesolimbic areas), via elevations in calcium concentration. This in turn increased the phosphorylation of the D3R, rendering it inactive and insensitive to cocaine-induced behavioral activation. Within the PFC, Lauzon et al. (2012) demonstrated that D4 receptor stimulation (another member of the D2R family) potentiated the emotional salience of normally non-salient fear-related associative memories via a CaMKII-dependent signaling mechanism, further suggesting an important link between associative memory formation and D2R-CaMKII-dependent signaling integration. Thus, given evidence for functionally dissociable roles for D1R/D2R transmission via downstream modulation of ERK/CaMKII signaling pathways, how might opiate exposure state regulate these pathways in the context of associative opiate-related memory formation?

## Opiate exposure state controls an ERK-CaMKII-dependent molecular memory switch in the basolateral amygdala-prefrontal cortical pathway

To examine the potential molecular substrates controlling the effects of chronic opiate exposure and withdrawal on BLA-dependent associative opiate reward memory formation, Lyons et al. (2013) performed a series of molecular and behavioral studies in rats investigating the effects of opiate exposure state on expression levels and functional roles of intra-BLA ERK 1/2 and CaMKII signaling pathways during associative opiate reward memory formation. Given previous evidence demonstrating a functional boundary between D1R vs. D2R transmission controlling opiate reward memory formation as a function of opiate exposure state (Lintas et al., 2011, 2012), the authors tested if intra-BLA D1R-dependent associative morphine reward memory may depend upon downstream phosphorylation of the ERK 1/2 pathway in previously opiate-naïve rats. In addition, the authors examined whether D2R-dependent memory formation may involve phosphorylation of downstream CaMKIIα signaling, in the opiate-dependent and withdrawn state of exposure. Consistent with these hypotheses, Lyons et al. (2013) reported that pharmacological intra-BLA inhibition of ERK, selectively blocked the acquisition of opiate reward memory formation in the opiate naïve state. In contrast, inhibition of the CaMKII signaling pathway blocked the formation of associative opiate reward memory only in the chronically exposed and withdrawn states. Similarly, the ability of intra-BLA D1R or D2R activation to potentiate opiate reward salience was mediated through the ERK signaling pathway in the naïve state, but switched to a CaMKII dependent pathway once rats were chronically exposed to opiates and in withdrawal (Lyons et al., 2013).

**Figure 5:**
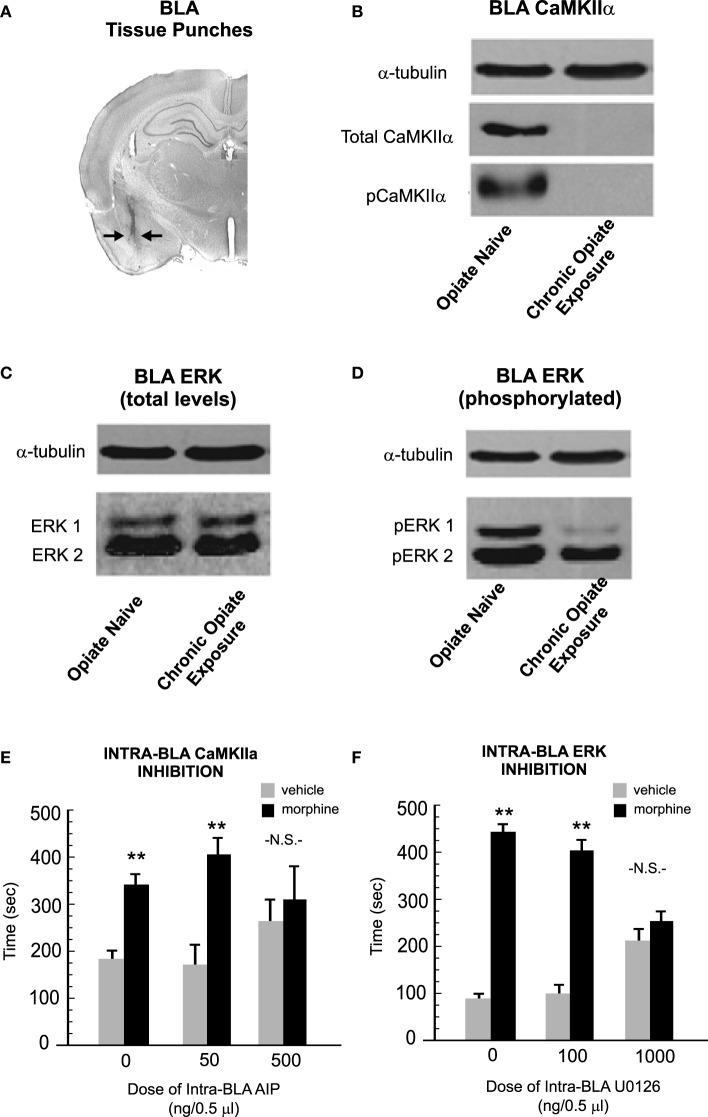
**Opiate exposure state controls the phosphorylation and functional roles of ERK 1/2 vs. CaMKIIα-dependent molecular memory substrates in the BLA**. **(A)** Using tissue punches from the BLA, Lyons et al. (2013) compared relative expression levels of CaMKIIα, ERK 1 and 2 total and phosphorylated levels across opiate-naïve vs. chronically exposed/withdrawn states. **(B)** Relative to opiate-naïve rats, chronic opiate-exposure and withdrawal completely abolished protein expression levels of both total and phosphorylated levels of CaMKIIα in the BLA. **(C,D)** In contrast, chronic opiate exposure and withdrawal selectively decreased levels of intra-BLA phosphorylated ERK 1. **(E)** Using a CPP paradigm, the acquisition of associative opiate reward memories is dose-dependently blocked by intra-BLA inhibition of CaMKII auto-phosphorylation with autocamtide-2-related inhibitory peptide (AIP), selectively in the chronic opiate exposure state. **(F)** In contrast, intra-BLA inhibition of ERK signaling with U0126, selectively and dose-dependently blocked opiate reward memory formation in the opiate-naïve state. ^**^p < 0.01. Figure Adapted from Lyons et al. (2013).

Concomitant with these behavioral dissociations, it was found that phosphorylation levels of both ERK 1/2 and CaMKIIα were dynamically regulated by opiate exposure levels. Thus, when relative protein expression levels of ERK 1/2 and CaMKIIα were measured in BLA tissue samples (Figure 5A) analysis of phosphorylated levels of CaMKIIα revealed a striking and dramatic reduction in total and phosphorylated levels of CaMKIIα (but not in other CaMK isoforms, such as CaMKIIβ; Figure 5B). In contrast, chronic opiate exposure and withdrawal led to a significant reduction in relative BLA expression levels of phosphorylated ERK 1 and 2 isoforms (Figures 5C,D). These intra-BLA molecular alterations corresponded to behavioral changes in sensitivity to intra-BLA blockade of CaMKII or ERK signaling, respectively. Thus, blocking intra-BLA CaMKII autophosphorylation blocked the acquisition of an opiate reward memory selectively in rats that were conditioned in a state of opiate dependence and withdrawal (Figure 5E). In contrast, blocking intra-BLA ERK signaling was only able to block opiate reward memory formation in rats conditioned in an opiate-naïve state (Figure 5F). Finally, as described previously, intra-BLA D1R or D2R activation with selective pharmacological activators has been demonstrated to potentiate the reward salience of morphine conditioning cues measured in a CPP paradigm (Lintas et al., 2012). In order to functionally link the effects of intra-BLA D1R vs. D2R transmission with ERK vs. CaMKII signaling mechanisms, Lyons et al. (2013) next demonstrated that intra-BLA ERK inhibition was able to block the opiate reward salience potentiation induced by D1R activation only in the opiate-naïve state. In contrast, inhibition of CaMKII selectively blocked the reward potentiating effects of intra-BLA D2R activation, in the chronically exposed and withdrawn exposure states.

Opiate exposure also results in alterations to the function and expression of D2Rs and CaMKII in the PFC, although in a manner opposite to that of the BLA. Rosen et al. (2015) reported that chronic exposure to heroin lead to an upregulation of the D2R and CaMKIIα, thus rendering opiate reward memory formation resistant to inhibition of either the D2R or CaMKII, which is otherwise sensitive to this blockade in the opiate naïve state. Interestingly, this same study also found that simultaneous contralateral inhibition of intra-BLA ERK1/2 and intra-PFC CaMKII was sufficient to block the acquisition of an opiate reward memory (Rosen et al., 2015). Unilateral inhibition of either of these substrates was insufficient to block reward memory formation, further pointing to the role of ERK and CaMKII in opiate reward memory processing in both the BLA and PFC.

In addition to their involvement in the processing of opiate-related reward memories across different stages of drug exposure, considerable evidence also implicates the ERK and CaMKII signaling pathways in the processing of opiate-withdrawal related associative memory formation. For example, using a naloxone-induced opiate withdrawal conditioned place aversion (CPA) procedure, wherein animals learn to avoid environments paired with the aversive effects of opiate withdrawal, Wang et al. (2015) reported that the extinction (unlearning) of naloxone CPA memories was correlated with increased phosphorylation of both the ERK and cAMP response element-binding protein (CREB) in the dorsal hippocampus and BLA. Similarly, it was reported that the extinction of associative opiate withdrawal memories involved alterations in the epigenetic control of brain-derived neurotrophic factor (BDNF) directly within the ventro-medial region of the rat PFC. Interestingly, this mechanism depended upon signaling via the ERK-CREB signaling cascade, through an NMDA receptor-dependent mechanism (Wang et al., 2015).

Together, these findings demonstrate that ERK and CaMKII-dependent memory substrates undergo profound adaptive changes during specific phases of the opiate addiction process and, within the BLA, are functionally linked to the modulatory roles played by intra- D1R vs. D2R signaling mechanisms. Furthermore, these studies underscore the importance of the brain's state of opiate exposure, serving as a functional boundary between separate associative memory substrates controlling the formation of opiate-related addiction memories.

## Summary and future directions

Increasing evidence is revealing the importance of selective molecular memory substrates within the BLA-PFC circuit as important underlying mechanisms controlling opiate-addiction related behavioral and neuronal plasticity. The overwhelming importance of memory plasticity during the development and maintenance of opiate addiction is demonstrated by these profound molecular adaptations. Indeed, the picture that is emerging from both clinical and animal-based studies suggests that the mammalian brain undergoes a dramatic transformation during the transition from a non-dependent to a dependent state, characterized by discrete and selective molecular and neuronal adaptations. Not only do critical neuroadaptations take place in terms of mediating the primary rewarding properties of opiates (Laviolette et al., 2004), but secondary, associative memory “switching” mechanisms occur in neural regions extrinsic to brain areas sub-serving opiate-related reward processing. Nevertheless, many critical questions remain. Most notably, how enduring are the observed molecular memory adaptations within the BLA-PFC circuit? How might the molecular adaptations observed within the BLA-PFC circuitry relate to other aspects of opiate-related memory processing and formation such as extinction and relapse phenomena? Of equal importance, there is currently a dearth of clinical research in human subjects examining the underlying molecular alterations in amygdalar and cortical regions induced by chronic opiate abuse. It is therefore of critical importance to verify that similar molecular adaptations take place in the human brain, particularly comparing the acute vs. chronic effects of opiate exposure on cortical and sub-cortical memory circuits. Beyond the roles of these molecular mechanisms in the process of opiate addiction, considerable evidence has also implicated the ERK and CaMKII signaling pathways as important modulators of other forms of addiction, including psychostimulants such as cocaine and amphetamine (Lu et al., 2005; Miller and Marshall, 2005; Valjent et al., 2006; Girault et al., 2007; Wang et al., 2012; Wells et al., 2012; Pizzo et al., 2014; Steinkellner et al., 2014). Thus, future research will be necessary to identify both the similarities and differences between how specific drug classes may specifically regulate these pathways in the context of addiction-related memory formation. Understanding these mechanisms will be helpful in identifying potential common mechanisms controlling the switch from the non-dependent to drug-dependent states across different drug classes and their associated receptor targets.

In summary, identifying and characterizing the neuronal and molecular events underlying the transition from the drug-naïve to dependent states both anatomically and pharmacologically, may offer the best hope for developing more effective pharmacotherapies aimed at preventing or reversing the effects of chronic opiate exposure and dependence. Indeed, the ability to target the molecular adaptations responsible for the persistence of drug-related associative memories may offer a novel and powerful approach to attenuating the power of drug-related associative memories over drug seeking and other compulsive behavioral manifestations of opiate addiction.

### Conflict of interest statement

The authors declare that the research was conducted in the absence of any commercial or financial relationships that could be construed as a potential conflict of interest.
